# The therapeutic potential of ultra-short-acting β-receptor antagonists in perioperative analgesic: Evidence from preclinical and clinical studies

**DOI:** 10.3389/fphar.2022.914710

**Published:** 2022-10-11

**Authors:** Linbin Fu, Buwei Yu, Zheng Li, Zhiheng Liu

**Affiliations:** ^1^ Department of Anesthesiology, Shenzhen Second People’s Hospital (Shenzhen Institute of Translational Medicine), The First Affiliated Hospital of Shenzhen University, Shenzhen, China; ^2^ Department of Anesthesiology, Ruijin Hospital, School of Medicine, Shanghai Jiao Tong University, Shanghai, China

**Keywords:** β-receptor antagonist, antinociception, cytokines, neurotransmitters, GPCRs, Sp5c

## Abstract

Perioperative multimodal analgesia can reduce the side effects of a high concentration of opioids, improving the comfort of the patient. However, insufficient analgesia of this model has prompted researchers to explore new adjuvant analgesics. Recently, an increasing number of studies have found a low-grade analgesic effect in the clinical application of ultra-short-acting β-adrenergic receptor antagonists, which are conventionally used as pharmacologic agents in the cardiovascular system. The mechanism by which ultra-short-acting β-antagonists exert antinociceptive effects has not been clarified yet. In this review, we intend to address its potential reasons from the side of neurotransmitters, inflammatory cytokines, and signaling pathways, providing theoretical proof for the application of β-adrenergic receptor antagonists in analgesia.

## Graphical Abstract

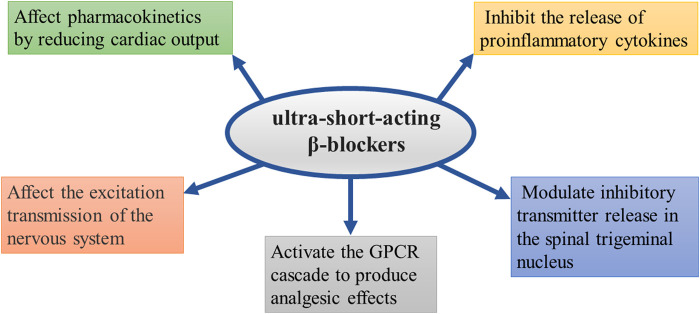

## Introduction

The International Association for the Study of Pain revised the definition of pain as “an unpleasant sensory and emotional experience associated with, or resembling that associated with, actual or potential tissue damage” (Raja et al., 2020). Surgery is a type of actual tissue injury, and postoperative pain is an important factor affecting patient recovery. Opioids can produce powerful analgesic effects, making them widely used in clinics for the treatment of perioperative and chronic pain (Tamara King, 2005). However, repeated administration of opioids can result in intraoperative acute, opioid-induced hyperalgesia, nausea, dizziness, and respiratory depression, significantly limiting extensive clinical use (Zhang et al., 2015; Ueda, 2004; Yu et al., 2016). Thus, multimodal analgesia, which can reduce the side effects of opioids and improve patient comfort, has developed as the main perioperative analgesia model (Gelman et al., 2018). Typical perioperative multimodal analgesics include opioids and nonsteroidal anti-inflammatory drugs (NSAIDs). However, NSAIDs are often associated with an increased risk of gastrointestinal, renal, and cardiovascular toxicities, thus limiting their application in patients with gastrointestinal and cardiovascular problems. The need for a new multimodal analgesic strategy that minimizes the side effects of opioids and improves the prognosis of the patient has become a serious clinical issue.

Ultra-short-acting β-blockers, including esmolol, landiolol, and flestolol, mainly act on myocardial β_1_ receptors but also block β_2_ receptors at high doses. These drugs are often used to treat angina pectoris, myocardial infarction, arrhythmia, hypertension, and other cardiovascular diseases (Gorczynski, 1985). Recently, many studies found that β-blockers administered intraoperatively may possess analgesic properties (Supplementary Table S1). Perioperative use of these drugs reduces the inhalation anesthetic and opioid dose requirement (Chia et al., 2004; Tamara King, 2005). Perioperative β-blockade in elderly surgical patients has some advantages, including maintained hemodynamic stability, improved physical sense of well-being, and decreased analgesic requirements (Zaugg et al., 1999). Studies investigating the antinociceptive effect of β-receptor antagonists have increased in number, but the role of β-receptor antagonists in the modulation of postoperative pain remains unclear. In this review, we summarize previous findings and review several possible mechanisms that may explain the clinical value of β-antagonists as analgesics.

## Beta blockers affect pharmacokinetics by reducing cardiac output

Cardiac output (CO) can affect liver blood flow, which in turn affects the metabolism of drugs with a high liver extraction rate (Weiss et al., 1996). Kurita et al. (2013) studied the effect of changes in CO on the plasma concentration of remifentanil under pseudo-steady-state conditions. The results showed that the plasma concentration of remifentanil was inversely proportional to cardiac output. CO is negatively correlated with the initial targeted concentration of intravenously administered drugs (Fagiolino, 2002). Birkholz et al. (2018) found that CO can influence the pharmacokinetics of sufentanil. They suggested that the dosage of opioids should be adjusted according to the case of changed CO to avoid inadequate drug effects or prolonged recovery. In addition, the decrease in CO can reduce the dosage of intraoperative fentanyl and remifentanil (Henthorn et al., 1992; Kurita et al., 2013). β-Adrenergic antagonists can slow down the heart rate and reduce the contractility of the heart muscle by inhibiting the β_1_ receptor. Afify and Andijani, (2017) demonstrated that the use of β-blockers enhanced the analgesic effect of morphine in a mechanism that involved decreased CO. This phenomenon has been confirmed in clinical studies: one report suggested that perioperative esmolol administration can reduce the intraoperative use of inhalation anesthetic and fentanyl and morphine consumption for the first three postoperative days (Chia et al., 2004). In addition, Morais et al. (2020) found that intraoperative esmolol can promote the reduction in pain intensity and the need for analgesic supplementation. In septoplasty surgery, the amount of remifentanil could be reduced when the heart rate was significantly decreased by esmolol (Gökçe et al., 2009). Thus, clinical research suggests that β-blockers can reduce the dose of perioperative opioids and intraoperatively inhaled anesthetics by reducing CO.

## Beta blockers inhibit the release of pro-inflammatory cytokines

**FIGURE 1:**
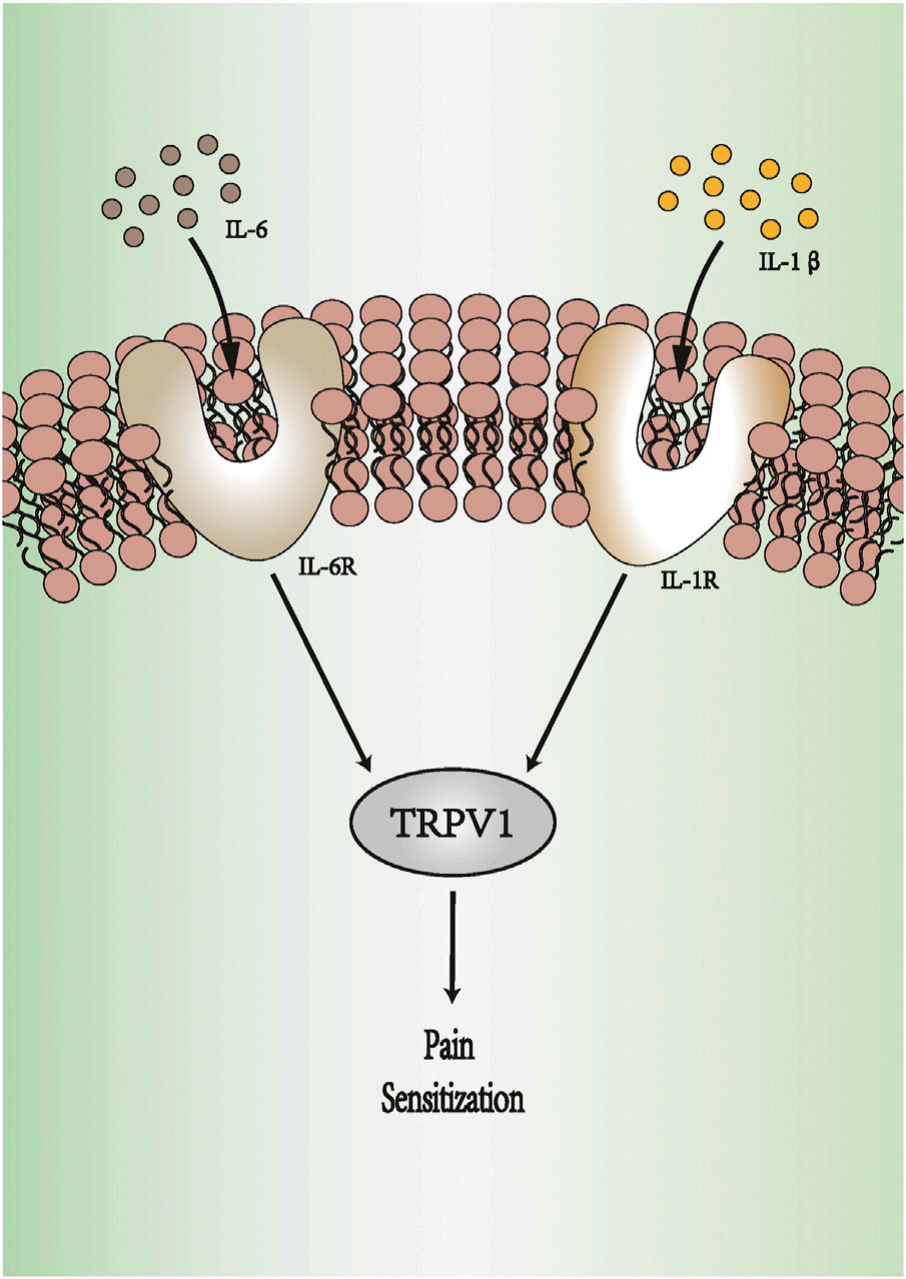
IL-6 and IL-1β produce pain sensitization. IL-1β binds to IL-1R, and IL-6 activates IL-6R. They can regulate pain by increasing the expression of TRPV1. The activation of TRPV1 increases action potential firing, heightening the sensitivity to pain. IL-1R: IL-1 receptor. IL-6R: IL-6 receptor. TRPV1: transient receptor potential vanilloid type 1.

Tissue damage, such as that from surgical incisions, can lead to inflammation and exacerbate the pain state of patients with pain (Brennan et al., 1996). This pain state results from the production and release of inflammatory mediators, such as prostaglandin E2 (PGE2) and pro-inflammatory cytokines. Pro-inflammatory cytokines are small molecular polypeptides synthesized and secreted by both immune and non-immune cells in the body. They regulate a variety of cellular physiological functions and play an important role under stressful conditions such as trauma, pain, and infection (de Oliveira et al., 2011). Examples of pro-inflammatory cytokines include interleukin (IL)-6, IL-8, and IL-13. These pro-inflammatory factors are released by glial cells or neurons and play important roles in the nervous system, participating in the development of acute or chronic pain (Cunha et al., 2005; Cunha et al., 2010). IL-1β and IL-6 have higher activity among the pro-inflammatory cytokines (Shyong et al., 2003) (Figure 1). Injecting IL-1β in the mouse abdominal region, ventricles, or plantar surface can cause hyperalgesia (Perkins and Kelly, 1994; Watkins et al., 1994). IL-1β can regulate pain by directly binding to transient receptor potential vanilloid type 1 (TRPV1) (Zhou et al., 2016). Phosphorylation of TRPV1 increases action potential firing, heightening the sensitivity to thermal or mechanical stimulation (Pinho-Ribeiro et al., 2017). In *in vivo*, the level of IL-6 is positively correlated with pain intensity and is related to the formation and development of neuropathic pain (Ramer et al., 1998; Yan et al., 2012). Malsch et al. (2014) suspected that IL-6 directly affects nociceptors in sensory neurons. The researchers decreased the pain threshold through the knockout of IL-6 receptors in sensory neurons. Intraplantar TNF-α was also reported to produce mechanical and thermal hyperalgesia induced by inflammation (Perkins and Kelly, 1994). TNF-α activates the p38 MAPK pathway in dorsal root ganglion (DRG) neurons to promote Na^+^ influx and reduce the excitability threshold (Jin and Gereau, 2006).

Activated β-receptors can increase the release of pro-inflammatory cytokines, such as IL-1β, IL-6, and TNF-α, and inhibiting these receptors can reduce the systemic and local inflammatory responses (Tan et al., 2021). Perioperative serum levels of IL-4 and IL-6 in patients undergoing laparoscopic gastrectomy were increased, and intravenous injection of esmolol reduced the increase of these inflammatory cytokines (Kim et al., 2015). Landiolol has also been shown to play a protective role in systemic inflammation by reducing serum levels of TNF (Hagiwara et al., 2009). Horikoshi et al. (2017) demonstrated that the application of landiolol can inhibit the increase of perioperative plasma IL-6 levels in patients undergoing esophageal cancer surgery. The pro-inflammatory cytokines such as IL-1β, IL-6, and TNF-α can increase the transmission of pain signals by reducing the action potential threshold. The inhibitory effect of β-blockers on the inflammatory response may alleviate their stimulating effect on perioperative pain.

## Beta blockers affect the excitation transmission of the nervous system

Nociception signals can transmit from primary afferent fibers to the central nervous system. The cell bodies of the primary afferent fibers responsible for the signal transmission are located in the DRG (Comitato and Bardoni, 2021). There are many excitatory ion channels on the membranes of DRG primary afferent fibers, such as voltage-dependent Na^+^, K^+^, and Ca^2+^ channels and the transient receptor potential channel. Blocking these channels in the DRG can reduce the transmission of pain signals and influence analgesia (Moravcikova et al., 2018; Hermanns et al., 2019). Peripheral nociceptors are stimulated to produce action potentials, and afferent fibers of the DRG transmit these signals to the spinal cord dorsal horn. The latter contains various subtypes of excitatory or inhibitory interneurons that can interact with each other to integrate pain-associated signals from primary afferent fibers and transmit these signals to neurons in the brain.

Tetrodotoxin-resistant sodium (TTX-rNa^+^) channels mainly exist in the DRG (Akopian et al., 1999). They can regulate the excitability of primary afferent neurons, influencing the transmission of nociception signals (Gold, 1999). Inflammatory mediators such as PGE2 continuously activate TTX-rNa^+^ channels through activation of the cAMP pathway and increase neuronal excitability (Li and Schild, 2007). IL-1β increases the excitability of pain receptors by alleviating the slow resting inactivation of the TTX-rNa^+^ channel and enhancing the persistent near-threshold TTX-rNa^+^ current (Binshtok et al., 2008). When several inflammatory mediators released in response to injury sensitize subpopulations of primary afferent neurons, blocking TTX-rNa^+^ channels can inhibit inflammatory mediator-induced modulation of current, indicating the important role of TTX-rNa^+^ channels in the treatment of inflammatory pain and hyperalgesia (Gold and Levine, 1996). In *in vitro*, esmolol can block the TTX-rNa^+^ channel current in a dose-dependent manner, and high concentrations of landiolol can also inhibit TTX-rNa^+^ channel activity (Tanahashi et al., 2009). These results suggested that β-adrenergic receptor antagonists can exert antinociceptive effects by blocking TTX-rNa^+^ channels. Lidocaine can bind to the S6 segment of domain 4 on the Na^+^ channel α-subunit in TTX-rNa^+^ channels (Tanahashi et al., 2007) and inhibit signal transmission. The electrophysiological characteristics of β-adrenergic receptor antagonists are similar to those of lidocaine (Wagner et al., 1999; Kim et al., 2000), which provides basic evidence for the local application of β-receptor blockers.

A report on *in vivo* administration of esmolol alone explored the effect of intrathecal administration in a postoperative pain model (Ono et al., 2015). The postoperative pain model was established with the use of a plantar incision. Withdrawal latencies were assessed by applying a focused radiant heat source to explore the antinociceptive effect of esmolol. The results suggested that plantar incision produced hypersensitivity in the postoperative pain model expressed as decreased withdrawal latency to heat stimulation. The decreased latencies caused by plantar incision were significantly increased by esmolol intrathecal administration after 5 min but not after 10 or 15 min. These results indicated that intrathecal administration of esmolol alone produced antinociceptive effects of short duration in a rat postoperative pain model. In addition, an inflammatory pain model was established with intraplantar injection of formalin. The expression of c-Fos (a marker of neuronal activation, including that induced by pain) was detected after formalin administration. The researchers found that intrathecal landiolol reduced the expression of c-Fos on the injection side, suggesting that intrathecal landiolol alone produces analgesic effects on inflammatory pain (Zhao et al., 2007; Mizobuchi et al., 2012). These studies indicate that intrathecal injection of β-receptor antagonists provides a new method to control perioperative pain. However, the clinical application and safety need to be further studied.

## Beta blockers activate the GPCR cascade to produce analgesic effects

G-protein-coupled receptors (GPCRs) comprise seven α-helical-structured transmembrane domains (TMs), an extracellular N-terminus, and an intracellular C-terminus (Gregory V Nikiforovich et al., 2007). In intracellular regions, GPCRs consist of heterotrimeric G-protein complexes, which contain Gα, Gβ, and Gγ subunits (Wess, 2022). According to their structure and function, Gα proteins are classified into four subfamilies (Gs, Gi/o, Gq/11, and G12/13) (Inoue et al., 2019). Different ligands acting on the extracellular side of GPCRs can change the TM and intracellular regions of the receptors (Wang et al., 2004). The effect on the latter may have different effects on downstream signal cascades. For example, Gs and Gi/o have opposite effects on adenylate cyclase (AC), which synthesizes cyclic adenosine monophosphate (cAMP) to activate protein kinase A (PKA) (Wess, 2022). Gs activates the process, and Gi/o inhibits the production of cAMP. The results of a human study showed that norepinephrine prolonged capsaicin-induced skin sensitivity (Drummond, 1995). In addition, Khasar et al. showed that adrenaline can cause skin mechanical hyperalgesia and sensitize DRG neurons. These effects of adrenaline are mediated by the PKA and PKC pathways.

**FIGURE 2:**
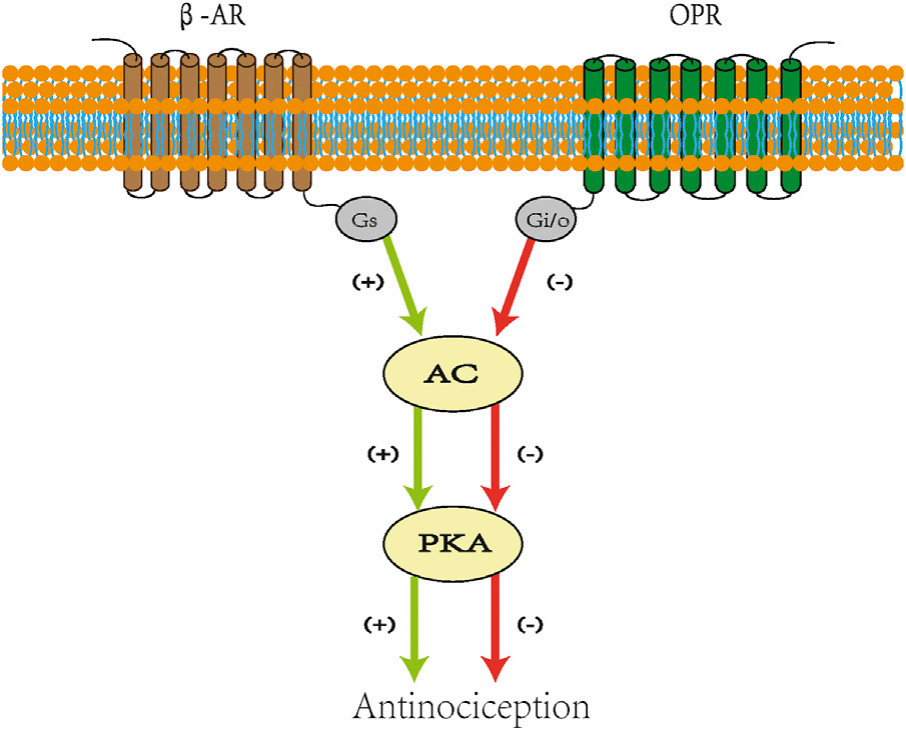
Scheme for crosstalk between opioid and β-adrenergic receptors. Stimulatory effects are indicated in green lines and inhibitory effects in red lines. Agonist acting on β-AR activates AC and stimulates PKA. Opioids combined with OPR inhibit AC and reduce the level of cAMP, which decreases the recruitment of PKA. β-Blockade prevents the stimulatory effect on AC enzyme and reduces the number of cAMPs. β-AR: β-adrenergic receptors. AC: adenylate cyclase. PKA: protein kinase A. OPRs: opioid peptide receptors. cAMP: cyclic adenosine monophosphate. Gs: stimulatory G protein. Gi: inhibitory G protein.

Studies show that the cAMP pathway may produce hyperalgesia and its inhibition can increase the pain threshold (Song et al., 2006; Shao et al., 2016). This finding means that the interaction between Gs and Gi/o directly affects the transmission of pain signals. Opioid peptide receptors (OPRs) and β-adrenergic receptors (β-ARs) belong to the GPCR family and can mediate similar cellular signal transduction cascades (Mitrovic et al., 2003; Zheng et al., 2010). β-ARs are Gs-coupled receptors whose stimulation promotes the activation of AC and PKA (Pepe et al., 2004). OPRs are Gi/o-coupled receptors whose stimulation inhibits AC activity to reduce PKA activation (Law and Loh, 1999). Research shows that β-blockade prevents the stimulatory effect on the AC enzyme, reduces the number of cAMP molecules, enhances the inhibitory effect of opioids on pain transmission, and provides an antinociceptive response (Afify and Andijani, 2017). These findings may be the result of Gs stimulation weakening the impact on Gi/o (Figure 2). This provides a basis for β-receptor blockers to enhance the analgesic effect of opioids.

## Beta blockers modulate inhibitory transmitter release in the spinal trigeminal nucleus

Facial somatosensory nerves project to multiple regions of the brain stem trigeminal nucleus complex (Fagiolino, 2002). This complex is divided into three regions that are, respectively, responsible for pain, touch, and temperature sensation. The spinal trigeminal nucleus (Sp5c), one part of the complex, is the primary site of reception and modulation of thermosensitive information. Its nociceptive signals arise from the cranio-orofacial regions. Sp5c has an important function in the modulation of nociceptive information (Takemura et al., 2006; Davies and North, 2009). In the resting state, neurotransmitters released by spontaneous exocytosis of single synaptic vesicles from the presynaptic membrane change the postsynaptic membrane current. Postsynaptic currents can be divided into miniature inhibitory postsynaptic currents (mIPSCs) and miniature excitatory postsynaptic currents (mEPSCs) according to the nature of the neurotransmitters that induced them (Chiang et al., 2021). Changes in these micro-currents also affect synaptic plasticity (Gonzalez-Islas et al., 2018). One report relayed the effect of esmolol’s role in Sp5c *in vitro* (Yasui et al., 2011). Brain stem slices containing Sp5c were placed in artificial cerebrospinal fluid with different Ca^2+^ concentrations. The role of Ca^2+^ in synaptic signal transmission was confirmed using esmolol intervention through recording mIPSCs and mEPSCs in synapses. The results showed that esmolol selectively increased the frequency of mIPSCs without affecting mEPSCs. It was suspected that esmolol produced analgesia *in vitro* by promoting the transmission of inhibitory signaling and reducing the transmission of nociceptive information to the nervous system. Esmolol increased the frequency of mIPSCs through a mechanism involving Ca^2+^ entry in a β-adrenoceptor-independent manner. The effect of β-receptor antagonists on pain signal transmission in Sp5c provides a new therapeutic strategy for the treatment of trigeminal neuralgia.

## Conclusion

At present, opioids still play an important role in clinical analgesia programs. However, there is an urgent clinical need to seek an ideal multimodal analgesia program that reduces the side effects of opioids and adjuvants while maintaining the ideal analgesic effects. Some clinical studies found that intravenous injection β-receptor blockers can reduce the consumption of opioids during the perioperative period and improve patient prognoses. This review discusses some potential analgesic mechanisms of β-blockers: 1) reduction of CO affects pharmacokinetics; 2) inhibition of the release of pro-inflammatory cytokines; 3) effects on the excitation transmission of the nervous system; 4) activation of GPCR cascades to produce analgesic effects; and 5) modulation of inhibitory transmitter release in the spinal trigeminal nucleus. Until now, only a few studies have found an effect of β-receptor blockers on analgesia. Landiolol and esmolol, two of the most studied ultra-short-acting β-receptor blocker drugs, have not been fully confirmed to act through the aforementioned possible mechanisms. In addition, animal experiments revealed that the analgesic effect lasted only a few minutes because the half-life of β-receptor blockers is short and the time for them to exert their analgesic effect is limited. However, the patient-controlled analgesia scheme will not limit their long-term clinical application. Although intravenously injected β-receptor blockers produce analgesic effects, clinical studies have also found that oral β-receptor blockers can reduce the dosage of opioid analgesics after the operation and reduce the adverse reactions caused by the latter. In addition, intrathecal injection of β-receptor blockers can also play an analgesic role by interfering with spinal cord neurotransmission. Perioperative multimodal analgesia is the basis for ensuring the safety of each combination of drugs and maximizing analgesic effects. Although preclinical and clinical studies have confirmed that β-receptor blockers can produce analgesia alone and assist other drugs in inducing analgesia, the mechanism of their specific participation in analgesia and the effectiveness of the route of administration still need to be further verified. In summary, this review provides evidence for the application of feasible alternative analgesics in perioperative multimodal analgesia.
