# Supplementary material for: The therapeutic potential of ultra-short-acting β-receptor antagonists in perioperative analgesic: Evidence from preclinical and clinical studies
Source: Front Pharmacol. 2022 Oct 11;13:914710. doi: 10.3389/fphar.2022.914710 (PMC9592752; doi:10.3389/fphar.2022.914710)
Supplement: Supplementary file 1 [file Table1.DOCX]

Supplementary Material

**Supplementary Table 1. Characteristics of clinical studies on the effect of intraoperative esmolol on intraoperative and postoperative analgesic consumption and others included in recent ten years.**

| **Type of Surgery** | **Esmolol Regimen** | **RESULTS** | | | **Reference** |  |  |
| --- | --- | --- | --- | --- | --- | --- | --- |
| Reduce intraoperative analgesic consumption | Reduce postoperative analgesic consumption | Other effects |  |  |  |  |  |
| Mastectomy | Esmolol 0.5 mg/kg 10 min before induction then 100 µg/kg/min |  | √ |  | (Mendonca et al., 2021) |  |  |
| Rhinoplasty | Esmolol 5-10 µg/kg/min |  | √ | It provides hemodynamic stability in first 3 hours. | (Vahabi et al., 2018) |  |  |
| Septorhinoplasty | Esmolol 0.5 mg/kg then 0.05 µg/kg/min | √ | √ |  | (Celebi et al., 2014) |  |  |
| Lower limb orthopedic surgery | Esmolol 0.5 mg/kg 30 min before induction then 5 µg/kg/min |  | √ | Delayed patient requests for analgesics. | (Haghighi et al., 2015) | |  |
| Arthroscopic shoulder surgery | Esmolol 1 mg/kg then 15 µg/kg/min | √ | √ | Relieved postoperative pain. | (Elokda and Nasr, 2015) |  |  |
| Inguinal herniotomy | Esmolol 0.5 mg/kg then 5-15 µg/kg/min | √ |  |  | (Kamal and Hassan, 2015) | | |
| Lower abdominal surgery | Esmolol 0.5 mg/kg 20 min before induction then 0.5 µg/kg/min |  |  | Relieved postoperative pain without increasing the risk of awareness. | (Bhawna et al., 2012) |  |  |
| Colectomy | Esmolol 0.5-1.0 mg/kg 5 min then 0.5-2.0 mg/kg/h |  | √ | Inhibiting the intubation response. | (Song et al., 2021) |  |  |
| LSG | Esmolol 0.5-mg/kg then 15 µg/kg/min |  | √ | Reduced pain intensity. | (Morais et al., 2020) |  |  |
| Laparoscopic gynecologic surgery | Esmolol 0.5 mg/kg then 30 µg/kg/min | √ | √ |  | (Hwang et al., 2013) |  |  |
|  | Esmolol 0.5 mg/kg then 30 µg/kg/min | √ | √ |  | (Moon et al., 2011) |  |  |
|  | Esmolol 1 mg/kg then 30 µg/kg/min |  | √ | It provides hemodynamic stability. | (Sultan, 2015) |  |  |
| LC | Esmolol 0.5 mg/kg then 10 µg/kg/min | √ |  | Reduced VAS score during the early postoperative period. | (Lee et al., 2014) |  |  |
|  | Esmolol 0.5 mg/kg then 5-15 µg/kg/min | √ |  | It has more effective analgesia compared with a combination of remifentanil-ketamine. | (Lopez-Alvarez et al., 2012) |  |  |
|  | Esmolol 1 mg/kg then 50 µg/kg/min |  | √ |  | (Akelma et al., 2014) |  |  |
|  | Esmolol 0.5 mg/kg before induction then 0.05 µg/kg/min | √ | √ | Reduced VAS scores in the early postoperative period and prolonged the time to first analgesia. | (Dhir et al., 2015) |  |  |

VAS: visual analogue scale. LSG: Laparoscopic gastroplasty. LC: Laparoscopic cholecystectomy

**References:**

AKELMA, F. K., ERGIL, J., ZKAN, D. & AKNC, M., & H.GüMü. 2014. A comparison of the effects of intraoperative esmolol and lidocaine infusions on postoperative analgesia. *Anestezi Dergisi,* 22**,** 25-31.

BHAWNA, BAJWA, S. J., LALITHA, K., DHAR, P. & KUMAR, V. 2012. Influence of esmolol on requirement of inhalational agent using entropy and assessment of its effect on immediate postoperative pain score. *Indian J Anaesth,* 56**,** 535-41.

CELEBI, N., CIZMECI, E. A. & CANBAY, O. 2014. [Intraoperative esmolol infusion reduces postoperative analgesic consumption and anaesthetic use during septorhinoplasty: a randomized trial]. *Rev Bras Anestesiol,* 64**,** 343-9.

DHIR, R., SINGH, M. R., KAUL, T. K., TEWARI, A. & OBEROI, R. 2015. Effect of intravenous esmolol on analgesic requirements in laparoscopic cholecystectomy. *J Anaesthesiol Clin Pharmacol,* 31**,** 375-9.

ELOKDA, S. & NASR, I. 2015. The effect of esmolol infusion as an adjunct to total intravenous anesthesia on the total anesthetic and analgesic requirements in arthroscopic shoulder surgery. *Ain-Shams Journal of Anaesthesiology,* 8.

HAGHIGHI, M., SEDIGHINEJAD, A., MIRBOLOOK, A., NADERI NABI, B., FARAHMAND, M., KAZEMNEZHAD LEILI, E., SHIRVANI, M. & KHAJEH JAHROMI, S. 2015. Effect of Intravenous Intraoperative Esmolol on Pain Management Following Lower Limb Orthopedic Surgery. *Korean J Pain,* 28**,** 198-202.

HWANG, W. J., MOON, Y. E., CHO, S. J. & LEE, J. 2013. The effect of a continuous infusion of low-dose esmolol on the requirement for remifentanil during laparoscopic gynecologic surgery. *J Clin Anesth,* 25**,** 36-41.

KAMAL, M. & HASSAN, M. 2015. Intravenous esmolol versus ropivacaine abdominal wound infiltration for postoperative analgesia after inguinal herniotomy: a randomized controlled trial. *Ain-Shams Journal of Anaesthesiology,* 8.

LEE, M. H., CHUNG, M. H., HAN, C. S., LEE, J. H., CHOI, Y. R., CHOI, E. M., LIM, H. K. & CHA, Y. D. 2014. Comparison of effects of intraoperative esmolol and ketamine infusion on acute postoperative pain after remifentanil-based anesthesia in patients undergoing laparoscopic cholecystectomy. *Korean J Anesthesiol,* 66**,** 222-9.

LOPEZ-ALVAREZ, S., MAYO-MOLDES, M., ZABALLOS, M., IGLESIAS, B. G. & BLANCO-DAVILA, R. 2012. Esmolol versus ketamine-remifentanil combination for early postoperative analgesia after laparoscopic cholecystectomy: a randomized controlled trial. *Can J Anaesth,* 59**,** 442-8.

MENDONCA, F. T., TRAMONTINI, A. J., MIAKE, H. I., SEIXAS, L. F., DE CARVALHO, L. S. F. & SPOSITO, A. C. 2021. Intra-operative esmolol and pain following mastectomy: A randomised clinical trial. *Eur J Anaesthesiol,* 38**,** 735-743.

MOON, Y. E., HWANG, W. J., KOH, H. J., MIN, J. Y. & LEE, J. 2011. The Sparing Effect of Low-dose Esmolol on Sevoflurane during Laparoscopic Gynaecological Surgery. *Randomized Controlled Trial,* 39**,** 1861-9.

MORAIS, V. B. D., SAKATA, R. K., HUANG, A. P. S. & FERRARO, L. 2020. Randomized, double-blind, placebo-controlled study of the analgesic effect of intraoperative esmolol for laparoscopic gastroplasty. *Acta Cir Bras,* 35**,** e202000408.

SONG, F., JIN, Y., LI, P., ZHENG, C. & ZHAO, X. 2021. Effect of Different Concentrations of Esmolol on Perioperative Hemodynamics and Analgesia in Patients Undergoing Colectomy: A Prospective, Randomized Controlled Study. *Drug Des Devel Ther,* 15**,** 5025-5033.

SULTAN, S. 2015. Effects of esmolol infusion on recovery profile and discharge from postanesthesia care unit after ambulatory gynecologic laparoscopic surgeries. *Ain-Shams Journal of Anaesthesiology,* 8.

VAHABI, S., RAFIEIAN, Y. & ABBAS ZADEH, A. 2018. The Effects of Intraoperative Esmolol Infusion on the Postoperative Pain and Hemodynamic Stability after Rhinoplasty. *J Invest Surg,* 31**,** 82-88.
